# Lipoprotein heterogeneity in persons with Spinal Cord Injury: a model of prolonged sitting and restricted physical activity

**DOI:** 10.1186/s12944-015-0084-4

**Published:** 2015-07-28

**Authors:** Michael F. La Fountaine, Christopher M. Cirnigliaro, Racine R. Emmons, Steven C. Kirshblum, Marinella Galea, Ann M. Spungen, William A. Bauman

**Affiliations:** Department of Veterans Affairs Rehabilitation Research & Development Service National Center of Excellence for the Medical Consequences of Spinal Cord Injury, James J. Peters Veterans Affairs Medical Center, 130 West Kingsbridge Road, Bronx, NY 10468 USA; Department of Medicine, Icahn School of Medicine at Mount Sinai, New York, NY USA; The Institute for Advanced Study of Rehabilitation and Sports Science, School of Health and Medical Sciences, Seton Hall University, South Orange, NJ USA; Department of Physical Therapy, School of Health and Medical Sciences, Seton Hall University, South Orange, NJ USA; Department of Kinesiology, William Patterson University, Wayne, NJ USA; Kessler Institute for Rehabilitation, West Orange, NJ USA; Department of Physical Medicine and Rehabilitation, Rutgers New Jersey Medical School, Newark, NJ USA; SCI Medical Service, James J. Peters VA Medical Center, Bronx, NY USA; Department of Rehabilitation Medicine, Icahn School of Medicine at Mount Sinai, New York, NY USA

**Keywords:** Cardiovascular disease, Insulin resistance, Lipids and lipoproteins, Nuclear magnetic resonance spectroscopy, Paraplegia, Special populations, Tetraplegia

## Abstract

**Background:**

Persons with spinal cord injury (SCI) often have low levels of physical activity, which predispose to increased adiposity and decreased high density lipoprotein cholesterol (HDL-C) concentrations, and, generally, normal low density lipoprotein cholesterol (LDL-C) concentrations. In spite of the mixed lipoprotein profile, the SCI population has been reported to have an elevated risk of cardiovascular-related morbidity and mortality. Nuclear magnetic resonance spectroscopy may permit a more precise quantification of lipoprotein particle (P) species, enabling a more accurate inference of risk for cardiovascular disease (CVD) in the SCI population.

**Methods:**

Fasting blood samples were obtained on 83 persons with chronic SCI and 62 able-bodied (AB) subjects. Fasting plasma insulin (FPI), triglycerides (TG), and P number and size of VLDL (very low density lipoprotein), LDL, and HDL subclasses were determined. AB and SCI subjects were stratified based on HDL-C (i.e., Low <40 and Normal ≥40 mg/dl): AB-Normal (n = 48), AB-Low (n = 14), SCI-Normal (n = 49), and SCI-Low (n = 34). Factorial analyses of variance were performed to identify group differences in lipoprotein measurements. Pearson correlations were performed between the number of P by lipoprotein subclass, size, FPI, and TG.

**Results:**

The SCI-Normal group was not significantly different from the AB-Normal group for body composition, FPI, TG or LP-IR and had negligible differences in the lipoprotein P profile, except for fewer number and smaller size of HDL-P. The SCI-Low group had a similar lipoprotein profile to that of the AB-Low group, but with a lipid P composition associated with a heightened atherogenic risk and greater tendency toward insulin resistance by the Lipoprotein-Insulin Resistance (LP-IR) score. In the SCI-Low group, the decreased number and reduced size of lipoprotein P were more prevalent and may be associated with increased waist circumference (i.e., abdominal adiposity), relatively elevated TG values (compared to the other subgroups), and an underlying subclinical state of insulin resistance.

**Conclusions:**

Prolonged sitting and restricted physical activity in individuals with SCI had the most profound effect on the HDL-C and its lipoprotein P subclasses, but not on LDL-C, however its P subclasses were also unfavorably affected but not to the same degree. The quantification of lipoprotein P characteristics may be a potent tool for the determination of risk for CVD in persons with SCI.

## Background

The lipoprotein profile is a common clinical measurement used to stratify risk for coronary artery disease (CAD). Outcomes from decades of population-based epidemiological research have provided optimal ranges for high- and low-density lipoprotein cholesterol concentrations (HDL-C and LDL-C, respectively), that when coupled with other health, behavior and lifestyle assessments serve to provide clinicians with an estimate of the potential burden of subclinical vascular atherogenesis. Nuclear magnetic resonance (NMR) technology has advanced the diagnostic precision for lipoprotein cholesterol measurement by providing the concentration of the number of HDL, LDL, and very low density lipoproteins (VLDL) particles (HDL-P, LDL-P and VLDL-P, respectively) for sub-particle classifications (i.e., particle sizes ranging from small to large) [1] and, thus, provide the ability to further differentiate the proportion of potentially atherogenic to potentially anti-atherogenic lipid particles in the circulation. The insight gained from combining lipoprotein cholesterol concentration with that of the lipoprotein particle number is anticipated to permit a more accurate estimate of risk for vascular events [2–4]. Such an approach should serve to more appropriately guide clinical intervention with pharmaceutical and/or lifestyle modification (i.e., physical activity, diet, smoking, etc.) to lower cardiovascular disease (CVD) risk.

Reduced physical activity has gained increased attention as a strong associate of obesity, insulin resistance, and dyslipidemia, and these disorders are being observed earlier in the lifespan and at a higher incidence in persons with sedentary lifestyles than in more active individuals [5, 6]. Engaging in regular physical activity has been shown to decrease the risk for CVD-related mortality by as much as 40 % [7]. According to a report by the National Institute of Disability and Rehabilitation Research on the total non-institutionalized population in the United States, 13.6 % (31.3 million) of respondents report some activity limitation due to chronic health conditions, 3.8 % (8.8 million) are not able to perform any major activity, and 5.9 % (13.6 million) are limited in the amount or kind of major activity [8]. Although considered a modifiable risk factor for CAD in the general population, the magnitude of physical activity required to achieve cardiorespiratory fitness and a clinically meaningful change in biomarkers of CAD is not attainable in those with a severe physical disability, such as with spinal cord injury (SCI). There are an estimated 238,000-332,000 persons with SCI living in the United States, with about 12,000 new cases presenting annually [9]. Advances in the acute and chronic medical management of persons with SCI have resulted in increased survival rates, leading to lifespans approximating that of the general population. In spite of improved survival outcomes, persons with SCI are faced with significant morbidity and medical challenges due, in large part, to the deleterious metabolic consequences of physical inactivity. After SCI, a significant adverse shift occurs in body composition and metabolism; there is an initial rapid, and then more insidious and progressive, lean tissue atrophy below the neurological level of injury [10–13], an increased adiposity [14] with an associated heightened prevalence of insulin resistance and disorders of carbohydrate metabolism (e.g., impaired glucose tolerance, and diabetes mellitus) than that reported in the general population [12, 15–18]. During the chronic phase of SCI, a characteristic dyslipidemia in the SCI population is well appreciated to occur, with mean serum HDL-C concentrations <40 mg/dl [19], and, occasionally, elevated mean serum triglyceride concentrations [20]. It should not be of surprise that CVD-related morbidity in persons with SCI occurs earlier in life, at a greater prevalence than that of the general population, and is the primary cause of death after the first year of injury [21–23].

Population-based studies are difficult to perform because of the low incidence rates for SCI. Evidence-based treatment targets used in the general population may be inappropriate to medically manage a chronically immobilized population. Additional relevant information is needed to generate a better understanding of CAD burden in individuals with SCI in order not to underestimate risk. Effective treatment to reduce risk of CAD is dependent on early and accurate diagnosis; NMR spectroscopy offers potent clinical insight to assess atherogenic risk. With consideration for the elevated risk of CVD-related morbidity in the SCI population that is imparted by prolonged sitting times and restricted physical activity, the use of findings from NMR methodology may serve to identify those with SCI who have an adverse lipoprotein profile by lipid particle size and number, and the presumed association with insulin resistance, and also provide security of knowing that others with SCI are at lower risk. This study will report values and associations among the lipoprotein subclasses for particle size and number in persons with SCI for HDL-C at or above and below 40 mg/dl, the threshold level that is appreciated to be an independent risk factor for CAD. Knowledge of these interrelationships and the influence of inactivity on lipid particle size and risk for CAD may serve to further alert the medical community to provide guidance and more appropriate treatment to those with SCI who are identified to be at greatest vascular peril.

## Methods

### Subjects

Eighty-three individuals with non-ambulatory chronic SCI (>6 months post-SCI) and 62 able-bodied individuals were recruited for participation from the SCI Service, outpatient clinics and hospital staff of the James J. Peters Veterans Affairs Medical Center, Bronx, NY, and the Kessler Institute for Rehabilitation, West Orange, NJ. Men and women between the ages 20 and 65 were considered for study eligibility as a control or SCI subject if they were free of acute medical illness (i.e., not receiving treatment for an active medical condition), without known diagnosed chronic illness (i.e., heart disease, pulmonary disease, diabetes mellitus), and had the capacity to provide informed consent. Women were excluded from consideration if they were pregnant. No subjects were taking medications with known effects on any of the study-related outcome measurements; this list may include, but was not limited to all classes of hypolipidemic agents, insulin/insulin-sensitizing agents, or hormone-replacement therapies. No individuals who participated in this study were engaged in competitive athletics/training at the time of their enrollment. The study was approved by the Institutional Review Boards of the two study sites. Written informed consent was obtained from each subject prior to study participation.

### Data collection

Subject demographic, medical history, anthropometrics and venous blood samples were obtained during a single study visit. All participants were required to complete an overnight fast prior to arriving at the testing center between 8 and 11 a.m. for study evaluation, which included venous blood collection for determination of the lipid profile, lipid particle number and size, glucose and plasma insulin concentrations. Analysis for the serum lipid profile [total cholesterol, triglycerides, HDL-C and estimated LDL-C] was determined in the General Chemistry Laboratory of the host institution using an ADVIA 1650 automatic chemistry analyzer following standard procedures recommended by the manufacturer (Bayer Diagnostics, Newbury, UK). The HDL-P, LDL-P and VLDL-P number and size were determined using automated NMR Lipoprofile 3 (LP3) lipoprotein particle analysis, by previously described methods (LipoScience, Inc., Raleigh, NC) [24]. From the LP3 technique, the following lipid particle number measurements were determined for total VLDL (VLDL-P), total HDL (HDL-P), total LDL (LDL-P), and for their respective Large and Small subparticle sizes. A proprietary algorithm was used to create the Lipoprotein Insulin Resistance (LP-IR) score (LipoScience, Inc., Raleigh, NC). The LP-IR score, which is a composite index based upon a proprietary algorithm (Liposcience, Inc., Raleigh, NC) derived from the number of Large VLDL, Small LDL, Large HDL and the size of the VLDL, LDL and HDL, ranges from 0 (most insulin sensitive) to 100 (most insulin resistant), and this index has been suggested to possess a stronger association to insulin resistance than any independent parameter alone [25, 26]. Blood samples for fasting plasma insulin (FPI) were batch processed in duplicate by radioimmunoassay using techniques previously developed by our laboratory [27]. Fasting plasma glucose (FPG) concentrations were performed on an automated glucose analyzer (YSI 2300 STAT Plus, YSI Life Sciences, Yellow Springs, OH).

### Statistics

For comparison, the AB and SCI groups were dichotomized by the fasting HDL-C concentrations: <40 mg/dl or ≥40 mg/dl, forming four subgroups: AB-Normal, AB-Low, SCI-Normal, and SCI-Low. The following were compared between groups using factorial analysis of variance: demographic characteristics; anthropometric measurements; FPG and FPI; lipoprotein profile (i.e., HDL-C, LDL-C, TC, and TG); lipoprotein particle measures (total particles for each subclass: HDL-P, VLDL-P, LDL-P; HDL, VLDL; and LDL-P sizes: Large and Small HDL, Large and Small VLDL, Intermediate LDL, Large LDL, and Small LDL); and LP-IR; a Bonferoni post-hoc test was used to further characterize significant group main effects. The LP-IR was plotted to demonstrate the respective group means and individual scores (performed with GraphPad Prism version 5.04 for Windows, GraphPad Software, San Diego, CA, USA). To facilitate our understanding of the observed lipid particle subclass sizes, separate Pearson correlations to FPI and TG were performed within each group using bivariate models. Pearson Chi-square Tests were performed to determine if the groups differed in the frequency of categorical outcome measurements. Statistical analyses were completed using IBM SPSS Statistics 21 (IBM, Armonk, NY, USA). An *a priori* level of significance was set at p ≤ 0.05.

## Results

Subject characteristics from each of the 4 study groups are provided (Table 1). The groups were matched for age and weight, but trended toward a significant group difference for height (p = 0.09) and body mass index (BMI) (p = 0.06). The SCI-Low group had significantly greater waist circumference (p < 0.05) and waist-to-height ratio (p < 0.05) compared to the AB-Normal group. Within the two SCI cohorts, there was a similar distribution of individuals with paraplegia (i.e., injuries to the thoracic or lumbar spine regions), complete sensorimotor injuries (i.e., absence of sensation or motor function below the neurological level of injury), and the duration of injury. No significant differences were identified between groups in the frequency of ethnicity (p = 0.13), gender (p = 0.21) and smoking status (p = 0.87). There was a significant group main effect for FPG (p < 0.0001) and a FPI (p < 0.03). Bonferoni’s post-hoc test revealed that the AB-Normal group had elevated higher mean FPG values compared to the SCI-Normal (p < 0.05) and SCI-Low (p < 0.05) groups, but the group means for FPG values were all within the normal range (Table 2).

**Table 1:** Characteristics of Study Cohorts by HDL Group

	AB-Normal	AB-Low	SCI-Normal	SCI- Low	p-value	Post-Hoc
n	48	14	49	34	-	
Age (yrs)	40.0 (11.4)	39.4 (12.3)	44.2 (11.5)	41.8 (11.4)	NS	
Height (m)	1.72 (0.09)	1.73 (0.06)	1.75 (0.09)	1.76 (0.09)	0.09	
Weight (kg)	82.8 (19.8)	87.3 (16.4)	78.3 (17.3)	87.0 (21.6)	NS	
BMI (kg/m^2^)	27.8 (5.5)	29.0 (4.6)	25.4 (4.5)	27.8 (5.3)	0.06	
Waist Circumference (cm)	90.0 (13.8)	96.2 (12.9)	96.4 (13.4)	102.2 (16.2)	<0.01	1
Waist to Height Ratio	0.53 (0.08)	0.55 (0.07)	0.55 (0.07)	0.58 (0.09)	<0.05	1
Paraplegia/Tetraplegia (n)	-	-	23/26	17/17	-	
AIS A/B/C (n)	-	-	28/7/14	22/4/8	-	
DOI (yrs)	-	-	17.7 (13.4)	14.2 (11.3)	NS	
Gender M/F (n)			40/9	31/3		
Ethnicity (n)						
Caucasian	30	7	28	20		
African American	9	1	12	4		
Hispanic	7	3	8	9		
Asian	20	3	1	1		
Smokers (n)	5	3	5	4		

Data are expressed as group mean (SD). *AB* able-bodied, *SCI* spinal cord injury, Normal: HDL-C >40 mg/dl; Low: HDL-C ≤40 mg/dl; *BMI* body mass index, *AIS* American Spinal Injury Association Impairment Scale, *DOI* duration of injury. P-values represent significant group main effects. Significant comparisons from post-hoc analyses: ^1^AB –Normal v. SCI-Low = p < 0.05

**Table 2:** Blood and Lipid Profiles by HDL Group

	AB-Normal	AB-Low	SCI-Normal	SCI- Low	p-value	Post-Hoc
Glucose (mmol/l)	4.9 (4.7, 5.1)	5.0 (4.6,5.5)	4.4 (4.1, 4.6)	4.3 (4.0, 4.6)	<0.0001	1,2
Insulin (mU/ml)	18.1 (13.2, 22.9)	27.5 (17.8, 37.1)	19.1 (14.3, 23.8)	27.6 (22.0, 33.2)	0.03	
Lipids						
Triglycerides (mg/dl)	91 (75, 107)	124 (94, 153)	109 (93, 125)	137 (118, 157)	<0.001	2
Total Cholesterol (mg/dl)	187 (176, 197)	178 (158, 196)	181 (170, 192)	166 (153, 179)	0.1	
LDL-C (mg/dl)	109 (100, 118)	117 (101, 133)	110 (101, 119)	104 (93, 114)	NS	
HDL-C (mg/dl)	59 (56, 62)	37 (31, 42)	49 (46, 52)	35 (31, 38)	<0.0001	1,2,3,4
NMR lipoprotein particle measures						
VLDL Particle Number (μmol/l)	53.4 (45.3, 61.7)	61.5 (46.3, 76.8)	56.4 (48.3, 64.6)	71.8 (62.1, 81.6)	<0.05	2
Large VLDL (μmol/l)	2.6 (1.4, 3.8)	4.3 (2.6, 7.1)	3.6 (2.4, 4.8)	5.5 (4.0, 6.9)	<0.05	2
Small VLDL (μmol/l)	31.8 (26.7, 37.0)	30.2 (16.6, 35.7)	32.9 (27.9, 38.1)	41.2 (35.0, 49.3)	<0.05	5
LDL Particle Number (nmol/l)	1088 (989, 1187)	1349 (1166, 1534)	1211 (1112, 1309)	1175 (1056, 1293)	0.08	
IDL (nmol/l)	89 (67, 112)	97 (55, 139)	111 (90, 132)	87 (62, 112)	NS	
Large LDL (nmol/l)	503 (444, 561)	372 (265, 478)	467 (409, 524)	390 (321, 459)	0.06	
Small LDL (nmol/l)	504 (421, 587)	887 (735, 1041)	633 (551, 715)	698 (599, 796)	<0.0001	2,3
HDL Particle Number (μmol/l)	35.6 (34.2, 37.0)	28.2 (25.6, 30.8)	31.1 (29.7, 32.5)	25.2 (23.5, 27.0)	<0.0001	1,2,3,6
Large HDL (μmol/l)	6.6 (5.9, 7.3)	2.1 (0.7, 3.4)	4.6 (3.9, 5.3)	2.4 (1.5, 3.3)	<0.0001	1,2,3,4,6
Small HDL (μmol/l)	16.7 (15.3, 18.1)	17.9 (15.2, 20.5)	15.8 (14.4, 17.2)	14.6 (12.9, 16.3)	NS	
VLDL Particle Size (nm)	45.7 (43.8, 47.7)	49.0 (45.4, 52.6)	47.8 (45.9, 49.7)	48.5 (46.2, 50.8)	NS	
LDL Particle Size (nm)	20.9 (20.8, 21.1)	20.3 (20.0, 20.5)	20.9 (20.8, 21.1)	20.6 (20.4, 20.8)	<0.0001	2,3,4,6
HDL Particle Size (nm)	9.2 (9.1, 9.3)	8.6 (8.4, 8.8)	9.0 (8.9, 9.2)	8.7 (8.6, 8.9)	<0.0001	2,3,4,6

Data are expressed as group mean (95 % CI). *AB* able-bodied, *SCI* spinal cord injury, Normal: HDL-C >40 mg/dl; Low: HDL-C ≤40 mg/dl; *NMR* nuclear magnetic resonance, *VLDL* very low density lipoprotein, *LDL* low density lipoprotein, *IDL* intermediate density lipoprotein, *HDL* high density lipoprotein. P-values represent significant group main effects. ^1^AB-Normal v. SCI-Normal = p < 0.05; ^2^AB-Normal v. SCI-Low = p < 0.05; ^3^AB-Normal v. AB-Low = p < 0.05; ^4^SCI-Normal v. AB-Low = p < 0.05; ^5^AB-Normal v. SCI-Low = p = 0.06; ^6^SCI-Normal v. SCI-Low = p < 0.05

Lipid concentrations and lipoprotein particle number and size are provided for each of the 4 study groups (Table 2). In general, the SCI-Normal and AB-Normal groups were very similar for lipid concentrations and lipoprotein particles, but the SCI-Normal group had a comparatively fewer number and smaller size of HDL-P. By study design, the SCI-Normal group had a higher HDL-C compared to the AB-Low group, and this favorable improvement was displayed in all other lipid and lipoprotein particle endpoints. The LDL-C values were statistically similar, across all 4 study groups with the values residing in an acceptable clinical range, and, as such, not requiring lipid-lowering treatment. The SCI-Low group had a more adverse lipid profile, including that of triglycerides, and lipoprotein particles compared to the AB-Normal group (Table 2), and tended to have fewer HDL-P number and smaller particle sizes of compared to its AB-Low group.

**Fig 1:**
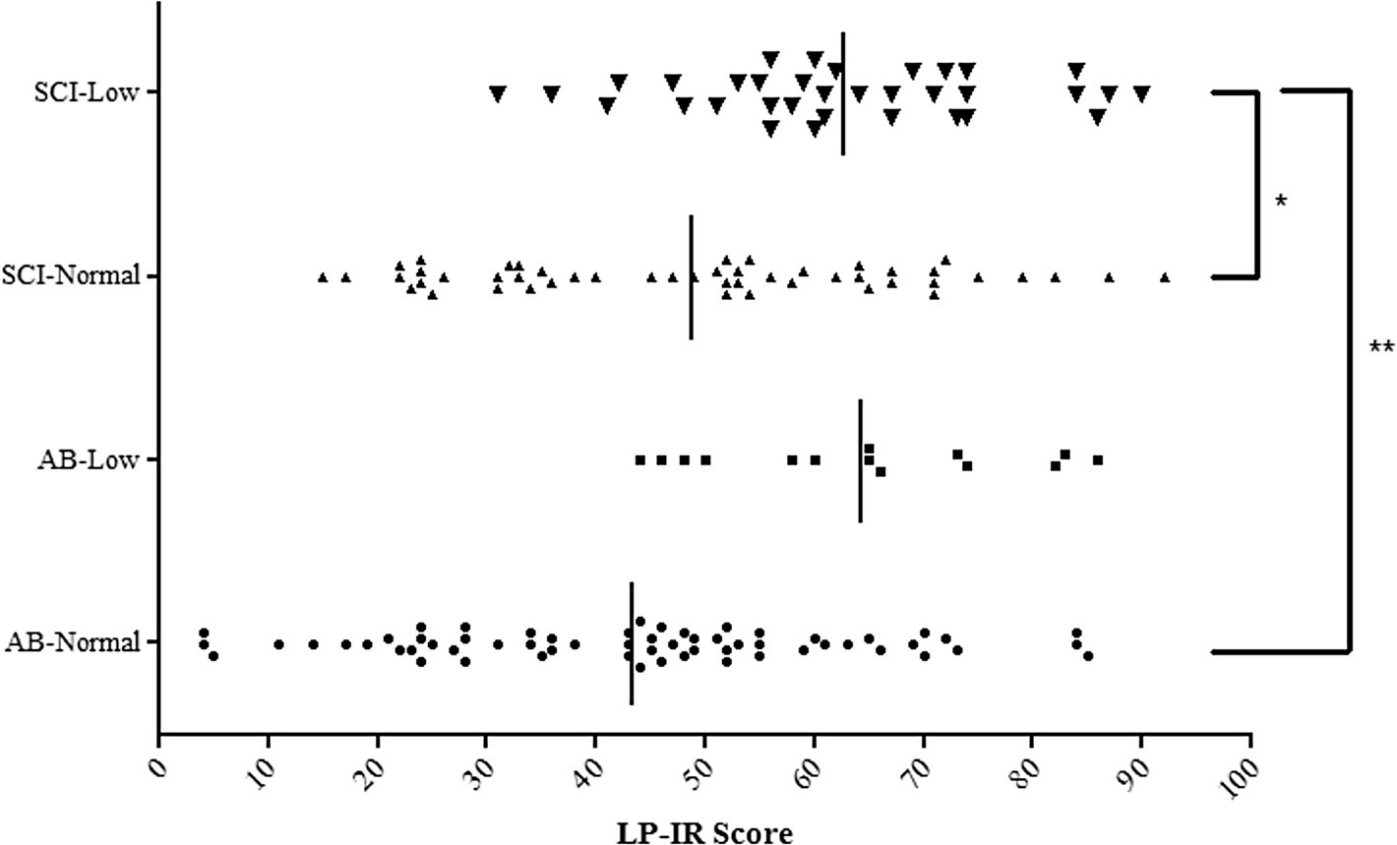
Lipoprotein Profile Insulin Resistance Score (LP-IR) across HDL groups. Data are presented as group mean (i.e., solid vertical bar) and individual subject score (i.e., small mark). *p < 0.01; **p < 0.0001. Abbreviations: AB = able-bodied; SCI = spinal cord injury; Normal: HDL-C >40 mg/dl; Low: HDL-C ≤40 mg/dl

Pearson correlations were performed between the respective number of lipoprotein particles by subclass, size, FPI (Table 3) and TG (Table 4). Except for the AB-Low group, FPI was the most consistently correlated (i.e., trended toward significance, or was significant) to the number of Small LDL-P and size of the VLDL particle in the other study cohorts. The SCI-Low group had a greater number of significant, or trends toward a significant, correlation between the respective sub-particles and FPI; these relationships were not unexpected because of the comparatively larger waist circumference observed in the SCI-Low group to those in the other groups, and thus the predisposition to insulin resistance. These significant relationships were supported by the LP-IR, where a significant group main effect (p < 0.0001) was observed and post-hoc tests revealed that the SCI-Low group had a significantly elevated LP-IR compared to the AB-Normal (p < 0.0001) and SCI-Normal (p < 0.01) groups (Fig. 1). In addition to FPI, TG concentrations had a demonstrable and deleterious influence on the lipid distribution (Table 4). Except in the AB-Low group, TG concentrations were significantly correlated to the number and size of lipoprotein subclasses. Increasing TG concentrations were positively associated with VLDL size and the number of Total and Large VLDL-P. TG concentrations were negatively associated with LDL size, but were positively associated to the number of Total and Small LDL-P. Increasing TG concentrations had no influence on Total HDL-P in all groups, but resulted in significant negative correlations for Large HDL-P in the SCI-Normal and SCI-Low groups; statistical significance was not achieved in the AB groups.

**Table 3:** Pearson correlations between NMR subclass particle size with FPI by HDL group

	AB-Normal		AB-Low		SCI-Normal		SCI-Low	
	r	p value	r	p value	r	p value	r	p value
Particle Size								
VLDL	0.27	0.09	-	NS	0.30	0.055	0.35	0.057
LDL	-	NS	0.6	0.09	-	NS	−0.32	0.09
HDL	−0.34	0.03	-	NS	-	NS	−0.31	0.09
Particle Subclasses								
VLDL								
Total			-	NS	−0.30	0.052	0.41	0.02
Large	0.27	0.09	-	NS	-	NS	0.36	0.053
Small	-	NS	-	NS	-	NS	0.33	0.07
LDL								
Total	-	NS	-	NS	-	NS	0.44	0.02
Intermediate	-	NS	-	NS	−0.27	0.08	-	NS
Large	-	NS	-	NS	-	NS	-	NS
Small	0.33	0.055	-	NS	0.32	0.04	0.60	0.0001
HDL								
Total	-	NS	-	NS	-	NS	-	NS
Large	−0.31	0.053	-	NS	-	NS	−0.37	0.05
Small	-	NS	-	NS	-	NS	-	NS

Correlations between group means for FPI and the respective NMR lipoprotein particle. *AB* able-bodied, *SCI* spinal cord injury, Normal: HDL-C >40 mg/dl; Low: HDL-C ≤40 mg/dl; *VLDL* very low density lipoprotein, *LDL* low density lipoprotein, *HDL* high density lipoprotein

**Table 4:** Pearson correlations between NMR subclass particle size with TG by HDL Group

	AB-Normal		AB-Low		SCI-Normal		SCI-Low	
	r	p value	r	p value	r	p value	r	p value
Particle Size								
VLDL	0.46	<0.001	0.64	<0.05	0.49	<0.0001	0.65	<0.0001
LDL	−0.27	0.06	-	NS	−0.50	<0.0001	−0.57	<0.001
HDL	−0.39	<0.01	-	NS	0.49	<0.001	−0.3	0.08
Particle Subclasses								
VLDL								
Total	0.75	<0.0001	0.88	<0.0001	0.59	<0.0001	0.56	0.001
Large	0.79	<0.0001	0.86	<0.0001	0.84	<0.0001	0.75	<0.0001
Small	0.37	<0.01	-	NS	-	NS	-	NS
LDL								
Total	0.36	<0.05	0.49	0.07	0.60	<0.0001	0.42	<0.02
Intermediate	-	NS	-	NS	-	NS	-	NS
Large	-	NS	-	NS	-	NS	-	NS
Small	0.32	<0.05	-	NS	0.70	<0.0001	0.66	0.005
HDL								
Total	-	NS	-	NS	-	NS	-	NS
Large	−0.25	0.08	-	NS	−0.37	0.01	−0.44	0.01
Small	0.26	0.07	-	NS	-	NS	0.39	0.02

Correlations between group means for TG and the respective NMR lipoprotein particle. *AB* able-bodied, *SCI* spinal cord injury, Normal: HDL-C >40 mg/dl; Low: HDL-C ≤40 mg/dl; *VLDL* very low density lipoprotein, *LDL* low density lipoprotein, *HDL* high density lipoprotein

## Discussion

Our report is the first to describe the adverse pattern of lipoprotein particle numbers and size in a highly sedentary population—that is, those with SCI who are characterized by obesity [14] and insulin resistance [12, 15–18], and in whom the risk for cardiovascular-related morbidity and mortality is elevated compared to the general population [21–23]. Our findings for atherogenic lipid patterns were then related to TG concentrations and degrees of insulin resistance, an underlying component of the metabolic syndrome that is becoming increasingly common across the lifespan in the general population. In this study, individuals with SCI and normal HDL-C (i.e., ≥40 mg/dl), who were not statistically different for body composition, FPI, TG or LP-IR from the AB-Normal group, had negligible differences in the lipoprotein particle profile (i.e., VLDL, LDL, TG, TC), except for HDL-C and the total number of HDL-P. However, the reduction in HDL-C and HDL-P in the SCI-Normal group may be speculated to be a consequence of greater degrees of insulin resistance due to the influence of unfavorable soft tissue body composition changes that occur with prolonged reductions in physical activity, characteristics which represent the classic determinants of insulin resistance [28]. Thus, it may be speculated that if individuals in the SCI-Normal group were to continue to gain adiposity, they would likely progress to an insulin resistant state and develop a lipoprotein profile comparable to those individuals in the SCI-Low group. The SCI-Low group (i.e., HDL-C <40 mg/dl), who were characterized as having an absolute or statistically significant increase in waist circumference, waist-to-hip ratio, TG concentrations and near significant elevations in FPI compared to AB-Normal and SCI-Normal groups, presented with a composition of lipid particles that is associated with a heightened atherogenic risk and greater tendency toward insulin resistance when characterized by the LP-IR. The SCI-Low and AB-Low groups were remarkably similar for many of the lipoprotein subparticles. Thus, those individuals with SCI who have an increased waist circumference (i.e., abdominal adiposity) and HDL-C <40 mg/dl may require a more aggressive level of evaluation and intervention; this may include the management of TG concentrations even if they are below the conventional therapeutic cutoff value to initiate pharmaceutical intervention. These points are especially relevant when considering that all LDL-C measurements were consistent across study groups and were well below therapeutic targets for initiation and management established by National Cholesterol Education Program Adult Treatment Panel III (NCEP ATP III) criterion [29].

As has been well appreciated, HDL-C concentrations are strong predictors for the risk of a cardiovascular event, even after adjusting for other conventional risk factors [30, 31]. More specifically, the risk for CAD increases by 3 % in women and 2 % in men for every 1 mg/dl decrease in HDL-C [32]. By this calculation of risk solely derived from HDL-C values, our SCI-Normal and SCI-Low groups have a minimum 20 % and 48 % increased risk for CAD, respectively, relative to our AB-Normal cohort. While our finding of depressed HDL-C tends to support the observed relative increase in cardiovascular-related morbidity and mortality rates in the SCI population [21–23], recent evidence from the Multi-ethic Study of Atherosclerosis (MESA) suggests that risk remediation had more to do with increasing the number of HDL-P than merely raising the HDL-C [3]. A reduction in the total number of HDL-P has predicted incident cardiac events in large and multi-national intervention trials [4, 33]. Increased numbers of HDL-P may reflect a greater capacity for reverse cholesterol transport to the liver for cholesterol disposal from the periphery [34] and possess a greater anti-oxidant capacity beyond that attributed to HDL-C alone [33], and thus be associated with a cardioprotective profile. Compared to the AB-Normal group, both the SCI-Low and SCI-Normal cohorts had markedly reduced numbers of Total and Large HDL-P, a lipid pattern which is compatible with heightened vascular risk.

The National Health and Nutrition Examination Survey (NHANES) revealed that increased sitting time was significantly associated with adverse cardiometabolic risk factors [35]; those individuals who had a self-reported time sitting for more than 6 hours per day, were more likely to have adverse FPI, HDL-C concentrations, and HOMA-IR and HOMA % B scores [36], and, thus, a greater inferred CVD risk. As a consequence of paralysis and the inability to assume upright standing and ambulation, time spent sitting in a wheelchair in those with SCI has been self-reported to be, on average, 9.2 hours per day [37], and, in the anecdotal experience of the authors, it is not uncommon for these individuals to spend in excess of 12–15 hours sitting in their wheelchair on a daily basis, with the remaining balance of non-sleeping time during the day spent in a seated or non-weight bearing position (i.e., seated on surface other than wheelchair). Our SCI cohort has lived with their immobilizing injury from 6 months to 47 years. Thus, the increased risk for CVD in the SCI population appears to be, in part, a consequence of chronic immobility, prolonged sitting and restricted physical activity, abnormal body composition and an associated greater prevalence of perturbations in carbohydrate and lipid metabolism [12, 14–18, 20].

Insulin resistance at the level of the adipocyte results in a state of relatively increased hydrolysis of triglycerides with the release of fatty acids into the circulation, stimulating the hepatic formation and secretion of VLDL [38] resulting in hypertriglyceridemia and a proatherogenic state [39]. According to presently accepted dogma, elevated circulating TG, mainly in the form of VLDL, deplete LDL-C and HDL-C by a two-step process: TG replaces cholesterol ester one-to-one in the lipid core through the action of cholesterol ester transfer protein, and then these incorporated TG in the lipid particles are hydrolyzed by the action of lipases, which results in cholesterol-depleted particles of reduced size across all subclasses [24, 40, 41]. The smaller LDL-P have the potential to migrate through the vessel wall, become more highly oxidized, and then more readily adhere to the subendothelial space; this sequence of events promotes ongoing atherogenic plaque formation. Insulin resistance may be postulated to have a direct adverse effect on the lipid subclasses and number of VLDL, LDL and HDL lipoprotein particles [25]; this concept was reinforced by the LP-IR score, which is a composite index of several of these particles, and was most adverse in the SCI-Low group.

Analogous to the prior discussion on HDL-P, the number and size of LDL-P are stronger predictors for CVD than relying exclusively upon LDL-C alone [42, 43]. As previously discussed, LDL-P possess a highly variable cholesterol content [40] with TG levels playing a determining role in species differentiation [24]. NCEP ATP III guidelines categorize LDL cholesterol levels for optimal, above optimal, borderline-high, and high/very high risk [29]. Similarly, the Framingham Offspring Study categorizes number of total LDL-P for optimal (<1100 nmol/l), above optimal (1100–1400 nmol/l), borderline-high (1400–1800 nmol/l), and high/very high (>1800 nmol/l) risk [44]. Employing this approach, the preponderance of our SCI cohort has a number of total LDL-P that fell within the near to or “above optimal” range and, as such, may certainly be candidates for appropriate intervention strategies if the lipid particle number is taken into consideration, but would not have been deemed to be suitable candidates for lipid-lowering therapy based on conventional approaches to care. Therefore, alternate strategies must be considered for the clinical management of the appreciated heightened risk of sub-clinical burden of vascular disease in this highly sedentary population.

In the general population, a clear association has been demonstrated between HDL-C and LDL-C [45, 46]. When HDL-C exceeds 50 mg/dl, Large LDL-P species predominate, but with values of HDL-C below 40 mg/dl, a striking increase in Small LDL-P occurs, associated with a precipitous decline in Large LDL-P [46]. As a result, there is a large disparity between LDL-C and LDL-P in persons with low HDL-C values, as was found in our SCI cohort. The marked reduction observed in the general population in large HDL-P at low HDL-C was also found to be evident to an even greater extent in our SCI-Low group. Thus, persons with SCI and low HDL-C, a designation which represents a far larger portion of the SCI than the general population, have an increased atherogenic risk not only on the basis of a depressed HDL-C, but also because of the adverse LDL particle profile, which may not be recognized to be a of concern if only the LDL-C is available when therapeutic decisions are being formulated.

In the literature, exercise and/or pharmacological interventions have been performed with success in the general population, and these life modifications have been performed to a far more limited extent in those with SCI. In persons with SCI, formally structured [47] and routine exercise programs [48], or the combination of exercise and diet programs [49] have demonstrated efficacy at modifying adverse lipid concentrations. Achieving a high-level of physical activity and performance serves to improve HDL-C compared to being sedentary in persons with SCI, but the restrictive effects of paralysis on physical activity favors a reduction of HDL-C compared to the general able-bodied population [50]. A randomized, double-blind, placebo controlled clinical trial with Niaspan was effective at increasing HDL-C by approximately 25 % in persons with tetraplegia, but, unfortunately, lipid particle number and size were not performed in this study [51]. Because of the limitations of physical activity in the SCI population, interventions with appropriate lipid-lowering agents may hold greater promise to promote vascular health. The LP-IR score displayed a vast distribution across the spectrum from insulin sensitivity to resistance, irrespective of HDL-C, possibly due in large measure to normal genetic variation of any population sample. The determinants of insulin resistance—that is, adverse soft tissue body composition and physical inactivity—are modifiable by lifestyle changes; thus, by increasing insulin sensitivity, the dyslipidemia may be anticipated to be mitigated, which ultimately would reduce atherogenesis and cardiovascular events. In individuals with non-modifiable limitations to activity, the ability to improve the determinants of insulin resistance by modification of lifestyle alone is severely compromised, leading to the appreciation that pharmacological intervention remains the only viable course of action to reduce CVD risk.

Our study design had limitations that precluded the ability to further differentiate risk or stratify outcomes in a descriptive manner, or to better identify the contributing factors for insulin resistance. Although persons with SCI have lower extremity paralysis that limits the intensity of exercise, individuals, especially those with lower cord lesions, may engage in varying degrees of physical activity as part of community mobility and leisure activity. However, the level of physical activity of our subjects with SCI was not captured, but the average intensity of activity may generally be assumed to be low because there were no SCI subjects capable of ambulation. Dietary habits and food choice is appreciated to play an integral role in determining the lipoprotein profile, but food diaries or dietary recalls were not obtained. Direct body composition measurements were not included in our analysis, which prevented us from determining if relationship(s) existed between total body or regional fat tissue compartments or lean tissue mass and lipoprotein particle number or size. Our subjects with SCI were community-dwelling individuals who frequented the two study sites, where they receive routine outpatient care or, on occasion, participated in clinical trials. Therefore, the results of the current report may be biased and may not reflect the larger SCI population, who are home-bound or do not routinely present themselves at medical facilities, or those who have little interest in participating in clinical investigation. Although there is confidence that our findings characterize the general SCI cohort, a larger, more diverse and comprehensive sampling from several geographical locations should be performed to confirm these results.

## Conclusions

The effects of restricted physical activity and adverse body composition changes in individuals with SCI had the most profound association to declines in HDL lipoprotein subclasses compared to the able-bodied groups. Once HDL-C concentrations fell below 40 mg/dl and associated with an increased waist circumference, a relatively elevated TG (compared to the other subgroups) and an underlying sub-clinical state of insulin resistance resulted; these factors were closely related to a more atherogenic profile of lipoprotein particles, despite most individuals with SCI having LDL-C values that failed to be in the treatment range to consider initiating lipid-lowering therapy. Therapeutic and lifestyle interventions to reduce CVD-related risk in the SCI cohort should be designed to reduce insulin resistance, reduce abdominal adiposity and, if possible, modestly increase the level of activity; more appropriate (i.e., lower) therapeutic thresholds may be considered for serum TG concentrations to improve the dyslipidemia associated with immobilizing disorders. Thus, the utilization of NMR spectroscopy to determine lipid particle number and size in persons who are extremely sedentary will provide clinical insight to more accurately predict the risk of CVD morbidity and mortality, and thus to prevent the underestimation of risk. NMR spectroscopy may also be utilized to determine the efficacy of clinical interventions which are employed to treat dyslipidemias in populations that are immobilized by neurological conditions or disease, spinal abnormalities, rheumatological ailments, and lower limb amputation in our effort to modify risk for CVD.
